# Predicting overall performance in Ironman 70.3 age group triathletes through split disciplines

**DOI:** 10.1038/s41598-023-38181-y

**Published:** 2023-07-17

**Authors:** Pantelis Theodoros Nikolaidis, David Valero, Katja Weiss, Elias Villiger, Mabliny Thuany, Caio Victor Sousa, Marilia Andrade, Beat Knechtle

**Affiliations:** 1 School of Health and Caring Sciences, University of West Attica, Athens, Greece; 2 Ultra Sports Science Foundation, Pierre-Benite, France; 3 Institute of Primary Care, University Hospital Zurich, Zürich, Switzerland; 4 Klinik Für Allgemeine Innere Medizin, Kantonsspital St. Gallen, St. Gallen, Switzerland; 5 Centre of Research, Education, Innovation and Intervention in Sport (CIFI2D), Faculty of Sport, University of Porto, Porto, Portugal; 6 Department of Health and Human Sciences, Loyola Marymount University, Los Angeles, CA 90045 USA; 7 Departamento de Fisiologia, Disciplina de Neurofisiologia e Fisiologia do Exercício, Universidade Federal de São Paulo, São Paulo, Brasil; 8 Medbase St. Gallen Am Vadianplatz, Vadianstrasse 26, 9001 St. Gallen, Switzerland

**Keywords:** Ageing, Epidemiology

## Abstract

Knowing which discipline contributes most to a triathlon performance is important to plan race pacing properly. To date, we know that the running split is the most decisive discipline in the Olympic distance triathlon, and the cycling split is the most important discipline in the full-distance Ironman^®^ triathlon. However, we have no knowledge of the Ironman^®^ 70.3. This study intended to determine the most crucial discipline in age group athletes competing from 2004 to 2020 in a total of 787 Ironman^®^ 70.3 races. A total of 823,459 athletes (198,066 women and 625,393 men) from 240 different countries were analyzed and recorded in 5-year age groups, from 18 to 75 + years. Correlation analysis, multiple linear regression, and two-way ANOVA were applied, considering p < 0.05. No differences in the regression analysis between the contributions of the swimming, cycling, and running splits could be found for all age groups. However, the correlation analysis showed stronger associations of the cycling and running split times than the swimming split times with overall race times and a smaller difference in swimming performance between males and females in age groups 50 years and older. For age group triathletes competing in Ironman^®^ 70.3, running and cycling were more predictive than swimming for overall race performance. There was a progressive reduction in the performance gap between men and women aged 50 years and older. This information may aid triathletes and coaches in planning their race tactics in an Ironman^®^ 70.3 race.

## Introduction

Triathlon is a multi-sports discipline that includes swimming, cycling and running^1^. Triathlon races are held over different distances: sprint distance (i.e., 750 m swimming, 20 km cycling, and 5 km running)^2^, Olympic distance (i.e., 1.5 km swimming, 40 km cycling, and 10 km running)^3^, half-distance Ironman^®^ triathlon (Ironman^®^ 70.3) (i.e., 1.9 km swimming, 90 km cycling, and 21.1 km running)^4^, full-distance Ironman^®^ triathlon (i.e., 3.8 km swimming, 180 km cycling and 42.195 km running)^5^, and ultra-triathlons (i.e., longer or multiple times the full-distance Ironman^®^)^6^.

In recent years, several studies attempted to define variables with an influence on triathlon performance for different race distances using various aspects such as laboratory analyses, training characteristics, previous experience^7,8^, psychological factors^9,10^ or anthropometric characteristics^11,12^. Other variables seemed predictive for the various split disciplines and race distances^2,7^. For example, for an Olympic distance triathlon, maximum aerobic velocity, triathlon experience, anaerobic threshold, and lean mass percentage were the most important variables in multiple regression models for performance estimation^7^.

Additionally, other studies investigated the most important split disciplines for various triathlon distances^13,14^. For the full-distance Ironman® triathlon, the cycling split was the most important split discipline^13,14^, whereas, for the Olympic distance triathlon, the running split seemed to be the most influential for overall race time^15^. Previous research showed that cycling was highly predictive for overall race time in Ironman^®^ 70.3 for professional triathletes^15^. However, there is no evidence which split discipline would be predictive in non-professional Ironman^®^ 70.3 triathletes (age group or recreational athletes)^16^. Furthermore, performance in the swimming split might influence performances in the other splits as it has been suggested by research on Sprint- and Olympic-distance formats. For instance, Peeling and Landers^33^ reported that a relatively low-intensity (without being slow) swimming split would result in faster cycling and running splits. Also, an analysis of the Olympic triathlon showed that competitive athletes should aim to complete each split as fast as possible and already from the swimming split to be in the “first pack”^9^.

The aim of this study was, therefore, to investigate which split discipline is the best predictor for overall race time in female and male Ironman^®^ 70.3 age group triathletes. There are differences in the age-related performance decline in Olympic distance triathletes compared to full-distance Ironman^®^ triathletes. The magnitude of the age-related performance declines in cycling and running performance is greater in the full-distance Ironman^®^ triathlon than in the Olympic distance triathlon^17^. Because the running split is the most influential split in the Olympic distance triathlon and the cycling split the most influential in the full-distance Ironman^®^ triathlon, we hypothesized that the running split would be the most influential in Ironman^®^ 70.3. It should be highlighted that the running split in the Ironman^®^ 70.3 (21 km) is closer to the 10 km of the Olympic distance triathlon compared to the 42.195 km in the full-distance Ironman^®^ triathlon.

## Materials and methods

### Ethical approval

This study was approved by the Institutional Review Board of Kanton St. Gallen, Switzerland, with a waiver of the requirement for informed consent of the participants as the study involved the analysis of publicly available data (EKSG 01-06-2010). All methods were performed in accordance with the relevant guidelines and regulations. The research has been performed in accordance with the Declaration of Helsinki.

### Data set and data preparation

The athletes' data were downloaded from the official Ironman^®^ website (https://ironman.com) using a Python script. The athletes' sex, age, country of origin, times for swimming, running, cycling, transition times 1 and 2, and overall race times were obtained. We analyzed successful age group finishers of all Ironman^®^ 70.3 races recorded on the Ironman^®^ website between 2004 and 2020^18^. Data considered for statistical analysis were the time of each split discipline of the Ironman^®^ 70.3 distance for swimming, cycling, running, and transition times (represented by transition 1—swimming to cycling and transition 2—cycling to running) and overall race times. Exclusion criteria were (i) athletes who did not start (DNS) or did not finish (DNF); (ii) disqualified athletes (DSQ); (iii) athletes with at least one missing split time; and (iv) inconsistent times (i.e., impossible split times, or final times smaller than split times, etc.).

### Groups stratification

Female and male athletes were divided into 5-year age groups: 18–24 years, 25–29 years, 30–34 years, 35–39 years, 40–44 years, 45–49 years, 50–54 years, 55–59 years, 60–64 years, 65–69 years, 70–74 years,75–79 years, 80–84 years and 85–89 years. Age groups 75–79 years, 80–84 years, and 85–89 years have been combined into a single group named '75 + ' (or just 75).

### Statistical analysis

The data distribution normality was validated by plotting the histograms of each split discipline and overall race times. Descriptive characteristics were presented as means and standard deviations, along with maximum and minimum values.

Correlation analysis (Pearson and Spearman) was performed to verify the association level between the overall race time and the split times (swimming, running, cycling and the first and second transitions), separately for males and females. Correlation coefficient classification criteria with 0.0 to 0.3 as a negligible correlation, 0.3 to 0.5 as a low correlation, 0.5 to 0.7 as a moderate correlation, 0.7 to 0.9 as a high correlation and 0.9 to 1.0 as a very high correlation were used^19^. Correlation matrixes were graphically presented for both sexes.

The overall race time is the sum of the partial times. This is deterministic, nonetheless, regressions can be built around the dataset. A multiple linear regression (MLR) was built to model the association between splits, transition times, and overall race times. Scatter plots were used to illustrate these associations.

The MLR model can be represented as follows:

$${\text{Finish Time}}\, = \,{\text{f}}\,\left( {{\text{ST}}, {\text{BT}}, {\text{RT}}, {\text{T1T}}, {\text{T2T}}} \right)\, = \,{\text{K}}\, + \,{\text{a}}*{\text{ST}}\, + \,{\text{b}}*{\text{BT}}\, + \,{\text{c}}*{\text{RT}}\, + \,{\text{d}}*{\text{T1T}}\, + \,{\text{e}}*{\text{T2T,}}$$

where: ST = Swim Time, BT = Bike Time, RT = Run Time, T1T and T2T are transition times and a, b, c, d, e are the regression coefficients and K the intercept constant.

Also, three univariate linear regression (ULR) models were built to try and find the "most predictive" split discipline.

$${\text{Finish}}\,{\text{Time }}\left( {{\text{Swim}}\,{\text{Time}}} \right)\, = \,{\text{S}}\, + \,{\text{s}}*{\text{ST}},$$

$${\text{Finish}}\,{\text{Time}}\,\left( {{\text{Bike}}\,{\text{Time}}} \right)\,{ = }\,{\text{B}}\,{ + }\,{\text{b*BT,}}$$

$${\text{Finish}}\,{\text{Time}}\,\left( {{\text{Run}}\,{\text{Time}}} \right)\,{ = }\,{\text{R}}\,{ + }\,{\text{r*RT,}}$$

where: S, B and R are the intercept constants of each model, s, b and r are the regression coefficients of each model, ST, BT and RT are the Swim Time, Bike Time and Run Time, respectively.

A two-way ANOVA was applied to compare the time performance for each age group for both female and male finishers. Results were considered significant at *p* < 0.05. Scatter plots and box plots were also used to illustrate the associations between split and overall race times and between sex and age groups. All statistical analyses were performed using the language Python and Google Colab notebooks.

### Institutional review board statement

This study was approved by the Institutional Review Board of Kanton St. Gallen, Switzerland, with a waiver of the requirement for informed consent of the participants as the study involved the analysis of publicly available data.

## Results

**Figure 1:**
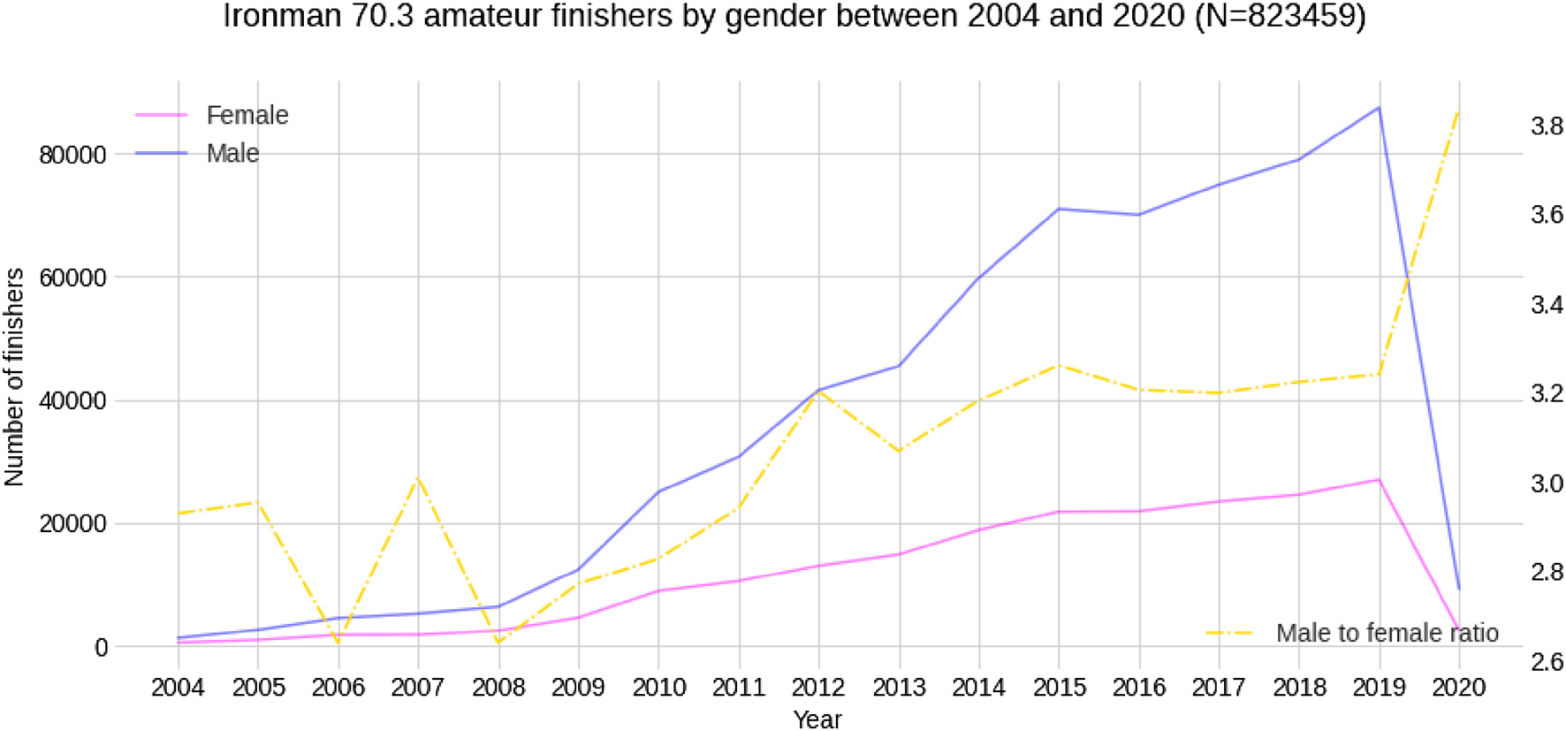
Number of finishers by gender and calendar year.

A total of 823,459 race records (198,066 from women and 625,393 from men) were finalists in Ironman^®^ 70.3 races from 2004 to 2020. Over the years, the number of recreational (age group) athletes increased where the male-to-female ratio remained at approximately 3.2 between 2012 and 2019, indicating a predominance of males (Fig. 1).

**Figure 2:**
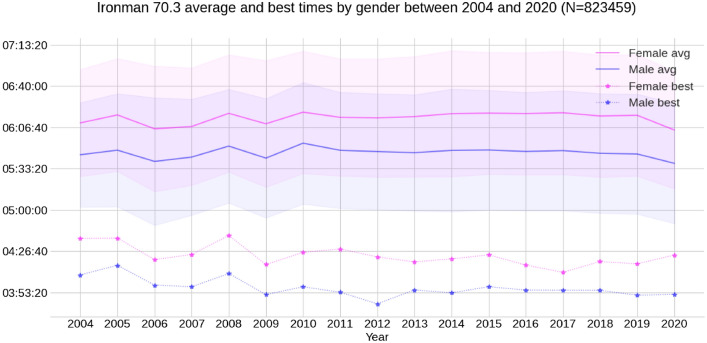
Average and best overall race times of all age group finishers by gender and calendar year. The shadowed areas represent the variance (standard deviation) of the average times.

**Figure 3:**
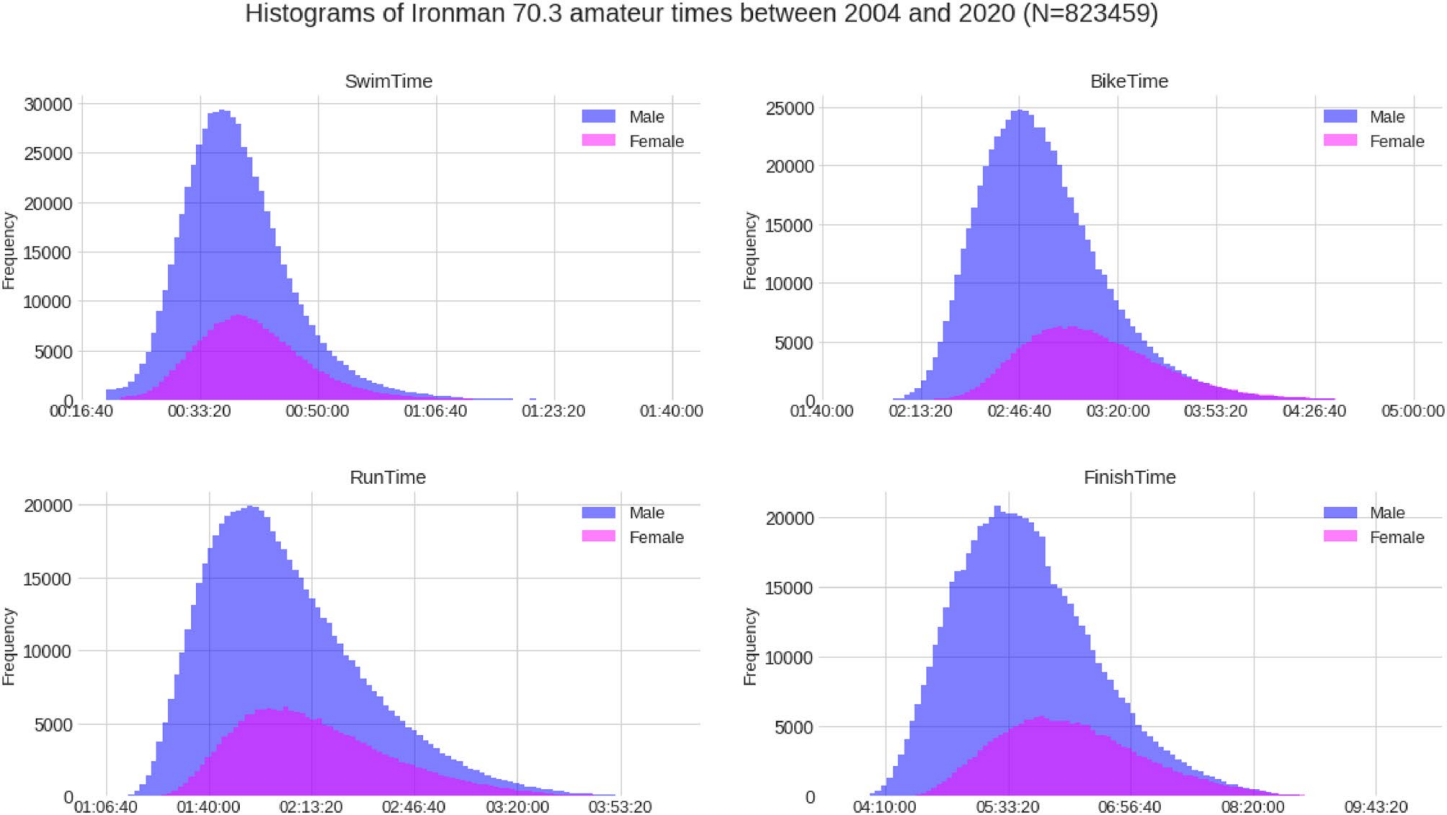
Distribution (histograms) of overall race times and split times by gender of all finishers.

The average and best finish times of all finishers by gender and calendar year are presented in Fig. 2. Women's best times decreased while men's best times remained flat. Average times have remained relatively flat for all whiles the number of participants soared. The distribution of finish and split times by gender is summarized in Fig. 3.

**Figure 4:**
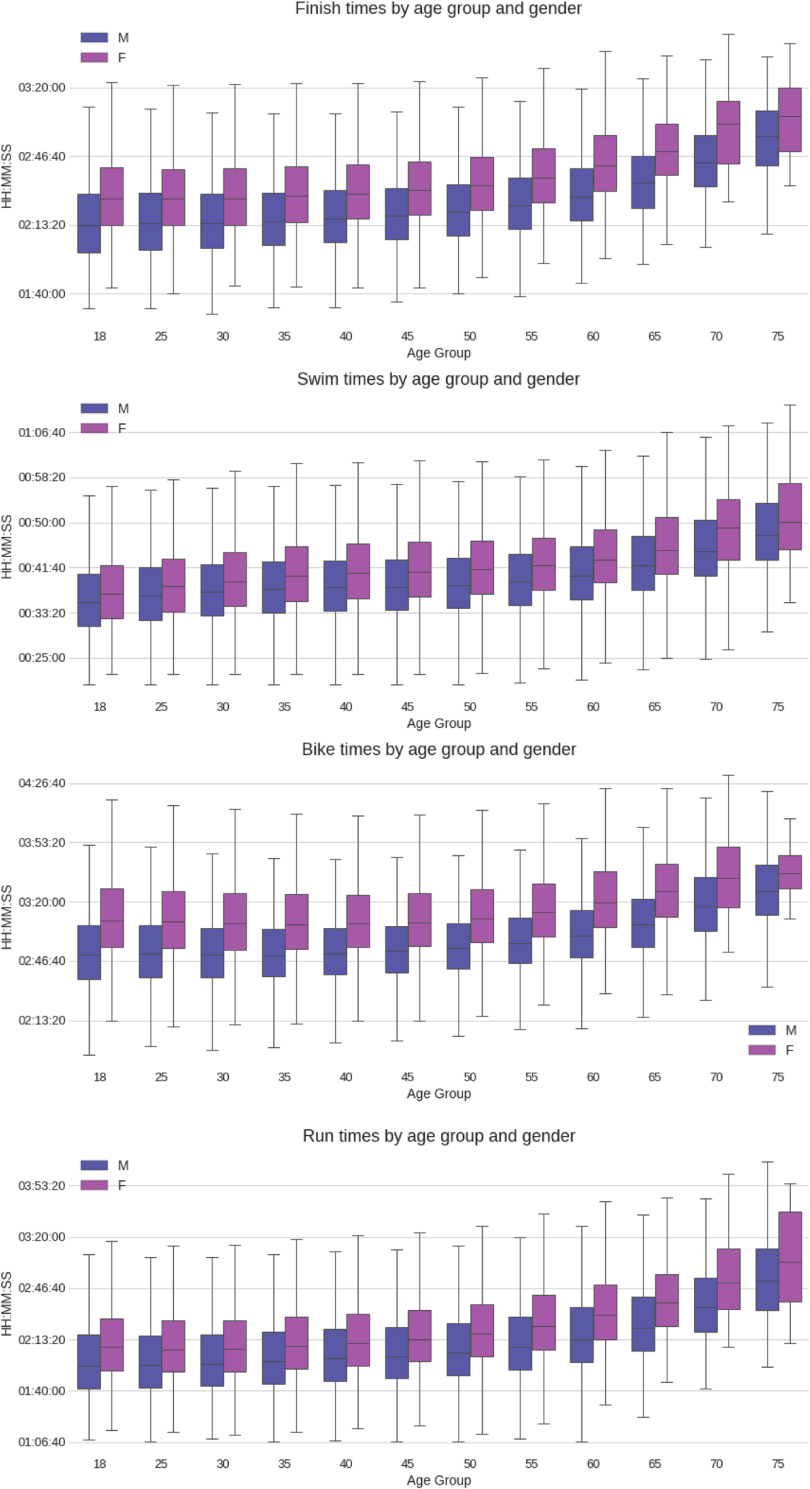
Finish and split times of all finishers by gender and age group.

Boxplots of split times and overall race times by gender and age group are presented in Fig. 4. The results of the two-way ANOVA tests applied to overall race times and split times showed that, for each main effect (*i.e.,* age group and gender) and their interaction the calculated *p*-values were zero or nearly zero. Therefore we rejected the null hypothesis and concluded that the differences between age groups and genders were statistically significant. For overall race times and split times, men were always faster than women in each age group (p < 0.05).

It is worth noticing how close male and female swim time boxes are, with significant overlap in the low and middle-aged groups compared to the bike or run splits (or to the overall race times). This suggests that the performance gap between men and women is smaller in the water (swimming) than on land (running and cycling). Bike times remained flatter than run and swim times through the low and middle-aged groups, suggesting the cycling split exhibits a more consistent performance across the different age groups.

**Figure 5:**
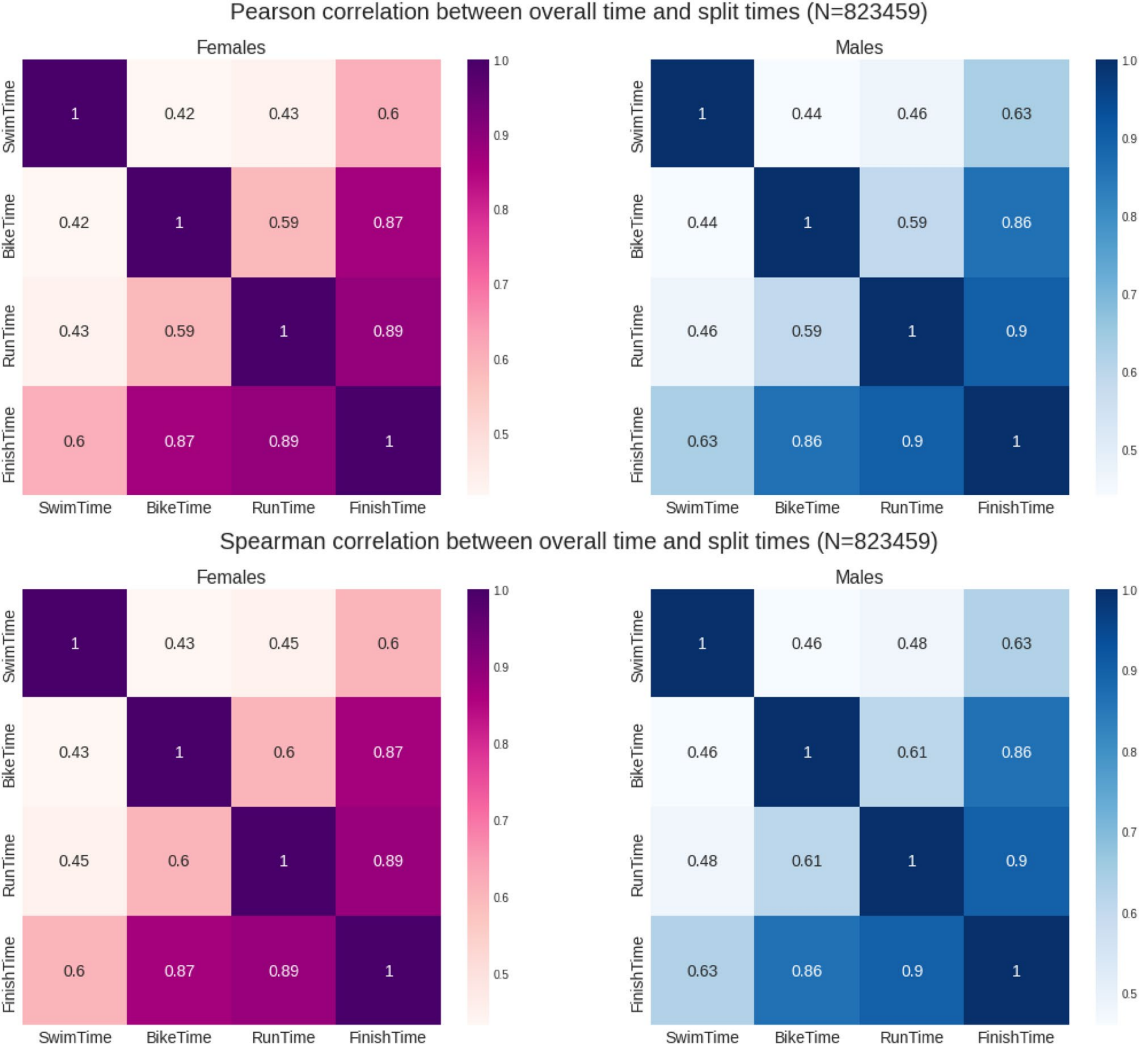
Correlation matrixes results between finish time and split times by gender of all age group finishers.

**Figure 6:**
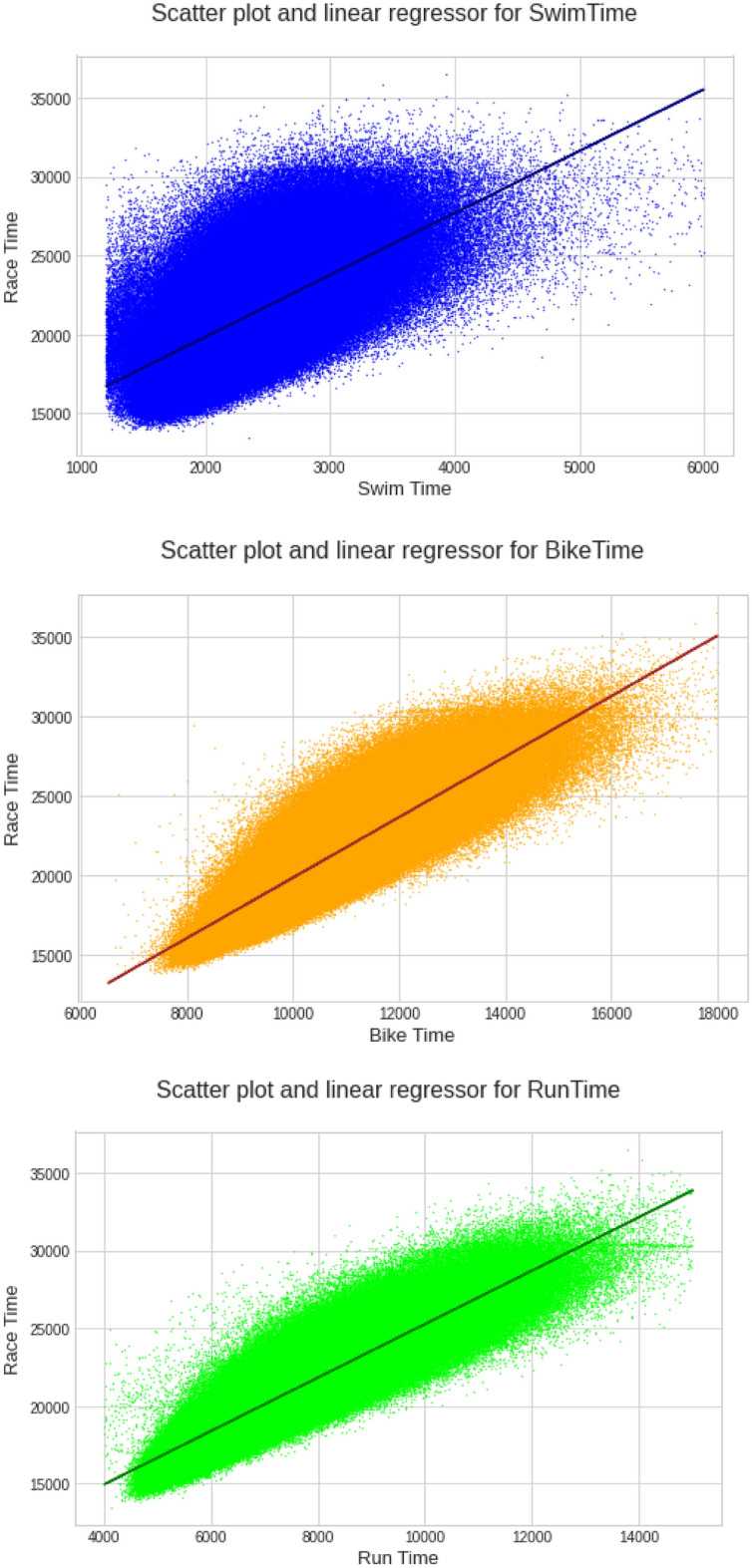
Univariate linear regressions (coefficients: m(swim) = 3.9283; m(bike) = 1.8996; m(run) = 1.7215).

Figure 5 presents the correlation matrixes between overall race time and split times. Running showed a positive and slightly higher correlation with overall race time (0.89 for women and 0.9 for men, moderate to high) than cycling (0.87 for women and 0.86 for men). When univariate linear regression models are calculated, the coefficients of bike and run times are much larger than the coefficient of swim time (0.394 for swimming, 0.759 for cycling and 0.805 for running), indicating their higher influence on the overall finish time (Scatter plots for each split discipline can be seen in Fig. 6). Figure 6 presents the scatter charts of the "Finish Time" versus split times showing the linear relationship with each variable.

**Figure 7:**
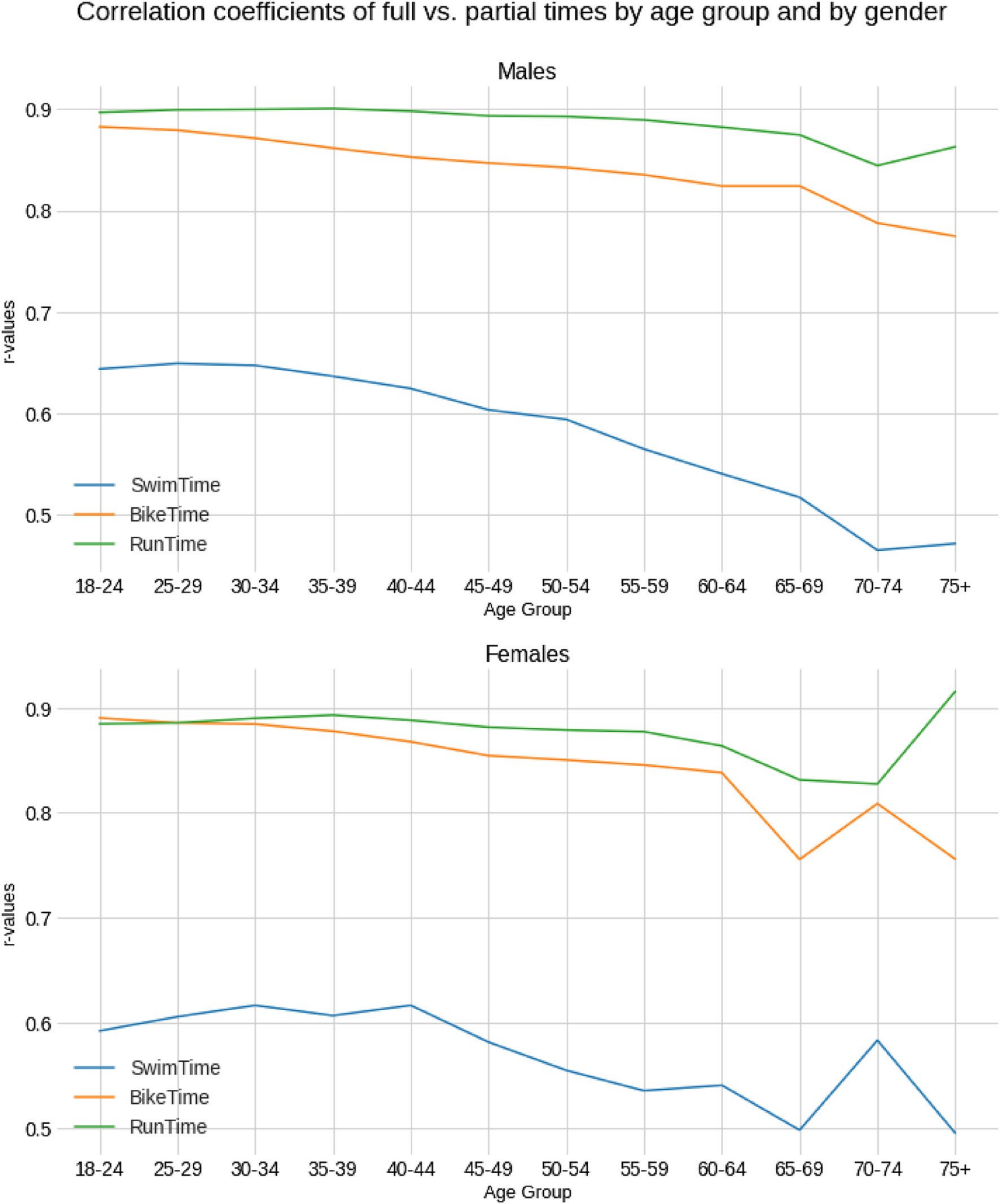
R-values for split disciplines for all age groups for both women and men.

Figure 7 summarizes the correlation coefficients (r-values) for women and men by age group. The swim correlation coefficients remain almost constant for females up to age group 40–44, before starting to decrease. For males. This decrease starts from age group 30–34. For the running and cycling disciplines, the correlation coefficients exhibit a smaller variation for both males and females.

## Discussion

This study investigated the best predictive split discipline in female and male Ironman^®^ 70.3 age group triathletes, hypothesizing that running would be the best predictor of the overall finish time. We found, however, no differences in the regression analysis between the contributions of the swimming, cycling, and running splits. The correlation analysis showed stronger associations of cycling and running with overall race time than swimming and a more negligible difference in swimming performance between males and females. The data also showed a progressive reduction of the performance gap between men and women in age groups 50 years and older.

The influence of split disciplines has mainly been investigated in the full-distance Ironman^®^ triathlon. The full-distance Ironman^®^ triathlon has a long tradition since 1978 with the first edition in Oahu, Hawaii^5^ [https://www.ironman.com/history]. Trends across years for the top three women and men competing in 'Ironman^®^ Hawaii' from 1983 to 2018 showed a significant decrease in each discipline for women and men, with cycling being the discipline with the most significant reduction. In addition, cycling was the discipline with the most significant influence on overall race time for both women and men^14^. For the full-distance Ironman^®^ triathlon, cycling was the most important split discipline^13,14^. When 51 male Ironman^®^ triathletes whom all finished a full-distance Ironman^®^ triathlon below 8 h were considered, cycling was the split discipline that predicted the best overall race time, followed by running and swimming. Furthermore, cycling was the discipline with the highest performance improvement over the years^13^.

For the top three women and men competing in the Ironman World Championship (Ironman^®^ Hawaii) from 1983 to 2018, cycling was the discipline with the strongest influence on overall race time for both sexes^14^. For the Olympic distance triathlon, however, the running split seemed to be the most influential in overall race time regardless of rank, position, or sex as shown in the data from 52,027 athletes who have competed in an official Olympic distance triathlon event (World Triathlon Series and Olympic Games) between 1989 to 2019^16^.

A potential explanation for the importance of the cycling split in the full-distance Ironman^®^ triathlon might be the age-related performance decline for the three triathlon split disciplines. The age of peak performance in the Ironman^®^ 70.3 was higher than in the Olympic distance triathlon and lower than in the full-distance Ironman^®^ triathlon^20^. A previous analysis of Ironman^®^ 70.3 performance showed that the decline of performance with age differed by the split, starting the earliest in swimming and later in running and cycling^21^. However, another analysis found an earlier performance decline in running than in swimming^22^.

It has been shown that the age-related decline in triathlon performance is specific to both the discipline and the distance, with cycling showing fewer declines in performance with age than swimming and running for both the Olympic distance triathlon, the full-distance Ironman^®^ triathlon^17^ and in ultra-distance triathlons^23^. For the Ironman^®^ 70.3 triathlon, the age-related decline started earliest in swimming (from the first age group on) for both men and women. For running, the performance decline began at 26 years in men and 28 years in women. The latest age-related decline started in cycling at 34 years for men and at 35 years for women^16^. Regarding the three split disciplines, swimming was generally less influenced for longer triathlon distances, such as an ultra-triathlon^6^.

We also need to consider the influence of the cycling split on the running split^24^. Under laboratory conditions, highly variable power distribution in cycling can impair the 10-km triathlon run performance^24^. Furthermore, differences have been reported regarding the cycling intensity of younger and older triathletes. It has been shown that elite master triathletes achieved a higher peak power output in cycling than elite junior triathletes^25^. Under field conditions, the different disciplines seemed to influence overall performance differently. A study investigating sprint distance triathlon, Olympic distance triathlon, Ironman 70.3^®^ (half-distance), and Ironman^®^ 140.6 (full-distance) showed that each split discipline had different importance in predicting overall race times regarding the length of a triathlon race. Swimming was the most predictive in sprint and Olympic distance triathlon, cycling in half-distance and running in full-distance Ironman^15^.

With regards to the variation of the relationship between overall and split times by age, we found a considerable decrease in the r-value for male swim times. This indicates to senior male competitors in Ironman 70.3 that, if they are aiming to improve the overall time, they need to train for a good bike and run times rather than for a fast swim split. The effect is not so noticeable in women, who managed to keep the swim split more relevant until the age group 40–44 years. In all cases, males and females, the run split show the highest value and flattest curve, so it is the unquestionable predicting factor from a Pearson correlation perspective. Compared to the other locomotion modes (running and cycling), swimming induces an increased workload on the upper limbs^26^; thus, it would be reasonable to observe a weaker relationship between swimming and overall triathlon performance, which relies on the lower limbs. The factors related to the decline in sports performance include both physiological and lifestyle changes^27^. Moreover, despite a general trend of a progressive age-related decline in world class performances in freestyle swimming, marathon running and triathlon^28^, it has been well known that muscle strength and power performances would exhibit a larger decline with aging than aerobic performances^29^. This observation was in agreement with an analysis of Olympic triathlon age group athletes at the World Championships, where a decrease of close to 5% per year after 45 years of age was observed in swimming, but a decline in performance was less pronounced in cycling until the age of 60 years^30^.

A limitation of the present study was the specificity of the triathlon format examined (Ironman^®^ 70.3) in terms of physiological and psychological demands^15,31,32^. Thus, caution would be needed to generalize our findings to other triathlon formats. Moreover, the number of finishers in the older age groups was smaller than that of younger age groups, which might explain some inconsistent results concerning differences between the oldest age groups (e.g. Figure 7). On the other hand, the strength of the study was that it analyzed a large database of Ironman^®^ 70.3 spanning 17 calendar years and including ~ 800 races and ~ 800,000 triathletes. These findings provide practical information for practitioners working with Ironman^®^ 70.3 athletes regarding setting optimal training goals based on sex, age and discipline. It should be noticed that the data analyzed concerned different races, which differed for slope, terrain, air and water temperature, wind and humidity. Thus, future studies should consider the role of these circuit characteristics and environmental conditions on the relationship of overall with split performances.

## Conclusions

Age group triathletes competing in Ironman^®^ 70.3 races have no differences in the regression analysis regarding the contributions of the swimming, cycling and running splits to overall performance. However, the correlation analysis showed stronger associations between cycling and running than swimming with overall race times. The present findings have practical applications for coaches working with triathletes preparing for Ironman^®^ 70.3. The knowledge that all disciplines were predictors of race performance would suggest that the training for this race should be balanced without neglecting any discipline. It was acknowledged that the cycling split was an excellent opportunity for fueling, and an optimal performance in this split would enable triathletes to cycle more comfortably at a higher exercise intensity (i.e., better exercise economy) and save energy for the running split.

## Data Availability

The athlete data was downloaded from the official Ironman website (https://ironman.com).
